# Scaffold-level validation is needed for phage–host inference from metagenome-assembled genomes

**DOI:** 10.1093/ismejo/wrag276

**Published:** 2026-11-04

**Authors:** Ling Yuan, Karthik Anantharaman, Bin Ma, Antonio Pedro Camargo, LinXing Chen

**Affiliations:** State Key Laboratory of Advanced Environmental Technology, School of Environment, University of Science and Technology of China, Hefei, Anhui, 230026, China; Department of Bacteriology, University of Wisconsin-Madison, Madison, WI 53706, United States; State Key Laboratory of Soil Pollution Control and Safety, Zhejiang University, Hangzhou, Zhejiang 310058, China; Zhejiang Provincial Key Laboratory of Agricultural, Resources and Environment, College of Environmental and Resource Sciences, Zhejiang University, Hangzhou, Zhejiang 310058, China; Department of Biochemistry, Institute of Chemistry, University of São Paulo, São Paulo, SP 05508-220, Brazil; State Key Laboratory of Advanced Environmental Technology, School of Environment, University of Science and Technology of China, Hefei, Anhui, 230026, China

**Keywords:** viruses, phages, phage–host relationship, genome binning, metagenome-assembled genomes

## Abstract

Accurate phage–host prediction underpins ecological interpretation and experimental validation of uncultivated phages, yet most current approaches rely heavily on metagenome-assembled genomes (MAGs). This reliance implicitly assumes that MAG-level quality metrics, such as completeness and contamination, ensure scaffold-level correctness. Here, we demonstrate that this assumption can be violated under particular challenging conditions. Tetranucleotide frequency (TNF) analyses reveal that CRISPR-associated regions often diverge compositionally from their host genomes, increasing the risk of misbinning, while experimentally validated phage–host genome pairs show consistent TNF separation between phages and their hosts, challenging compositional binning assumptions. Through controlled simulations, we further show that the incorporation of substantial amounts of TNF-matched, non-host phage sequences into high-quality MAGs does not substantially alter standard completeness and contamination estimates, rendering such errors largely undetectable. Together, these results expose a systematic and underappreciated source of bias in MAG-based phage–host inference and highlight the need for explicit scaffold-level validation beyond conventional genome-quality metrics. We further propose a practical scaffold-level validation framework that integrates structural linkage and taxonomic consistency to improve the reliability of phage–host inference from MAGs.

Accurate host prediction is not only central to the ecological interpretation of phages but also plays a critical guiding role in their isolation, cultivation, and experimental characterization [1], yet the hosts of most uncultivated phages remain experimentally unknown [2]. Computational approaches therefore infer host identity from genome sequence data using CRISPR spacer matching, prophage detection, sequence homology, compositional similarity, and integrated machine-learning frameworks [3–5]. Many of these approaches rely directly or indirectly on metagenome-assembled genomes (MAGs), which provide genome representations of bacteria and archaea in public databases [6–10]. This widespread reliance on MAGs implicitly assumes that MAG-level genome quality metrics (primarily completeness and contamination) are sufficient to ensure the correctness of host prediction at the scaffold level. However, these metrics were designed to evaluate recovery of cellular genomes using conserved marker genes [11–13], not to determine whether CRISPR-associated or viral scaffolds have been assigned to the correct host MAGs.

This distinction is important because most MAGs are composites of multiple scaffolds grouped by tetranucleotide frequency (TNF) and sequencing coverage profiles (Fig. 1a). Scaffolds of mobile genetic elements are particularly prone to misbinning in short-read metagenomic assemblies [14, 15], and MAG-based host inference is vulnerable in at least two common scenarios. First, the bacterial host of a viral scaffold may be predicted as the taxon of a MAG because it matches CRISPR spacers detected within that MAG. However, a scaffold containing only a CRISPR repeat–spacer array, or lacking sufficient host genomic context, may not originate from the organism represented by the MAG (Fig. 1b). Second, viral scaffolds detected within MAGs, or viral sequences homologous to MAG scaffolds, are often used for host assignment. Such evidence is reliable only when supported by integration signals, host–virus junctions, or sufficient host-flanking sequence. Otherwise, the viral scaffold may simply be co-binned into an unrelated host MAG (Fig. 1c). Importantly, the standard completeness and contamination estimates of MAGs cannot distinguish these cases.

**Figure 1:**
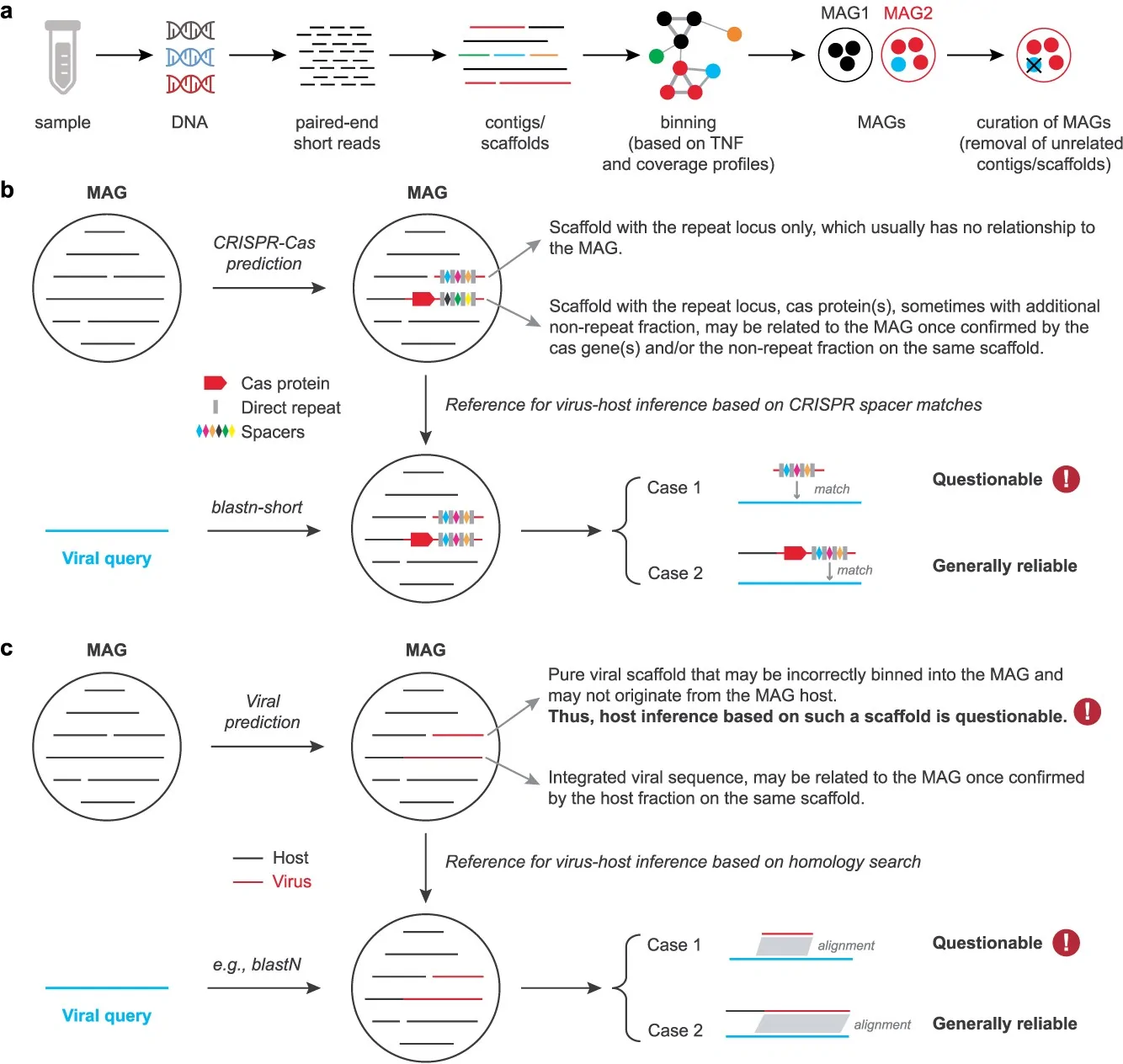
Concept illustration of the common pitfalls in using MAGs to infer phage-host relationships. (a) General workflow of metagenomic analyses leading to the reconstruction of metagenome-assembled genomes (MAGs). The curation of MAGs represents a critical step for removing unrelated contigs or scaffolds. However, this step is frequently omitted in routine analyses. (b) A MAG may contain a scaffold with only the CRISPR repeat locus region (repeats + spacers), which may be from another unrelated MAG. For a scaffold with a CRISPR repeat locus, cas gene(s), and additional host fraction, the longer the host fraction is, the more reliable the confirmation can be. (c) Viral scaffolds in MAGs may have no relationship to each other. For an integrated viral sequence, the longer the host fraction is, the more reliable the confirmation could be. In the metagenomic analyses based on short-read sequencing and assembly, the assembled scaffolds are generally binned to obtain MAGs based on their TNF and/or sequencing coverage profiles. However, infecting viral scaffolds and CRISPR repeat-array regions may have TNF and/or coverage profiles inconsistent with those of the host genomes.

We first tested whether CRISPR-associated regions have sequence compositions consistent with their host genomes. From 271 single-scaffold MAGs in the HRGM2 human gut genome collection [9], we identified 48 putative CRISPR–Cas systems and compared the TNF profiles of the whole genomes against the full CRISPR–Cas regions, cas gene regions, and CRISPR repeat arrays. Our analyses showed these CRISPR-associated sequences, especially repeat–spacer arrays, frequently occupied TNF space distinct from their corresponding host genomes and, in some cases, were compositionally closer to sequences from different phyla than to their own genomes (Supplementary Fig. S1). The same phenomenon was observed at the genome level for those from the phyla Firmicutes_A and Pseudomonadota (Supplementary Fig. S2). This pattern may be due to rapid spacer turnover, repeat enrichment, and the horizontal transfer of CRISPR–associated genomic fragments [16, 17]. It indicates that CRISPR-containing scaffolds can be compositionally atypical and could be co-binned into incorrect MAGs. Such errors can directly produce false-positive host links when MAG-derived CRISPR spacers are used for phage–host prediction.

We next asked whether phage genomes are expected to share similar TNF profiles with their confirmed hosts. Using experimentally validated host–phage genome pairs from *Streptomyces* [18] and *Escherichia coli*, we found that phage genomes generally separated from their bacterial hosts in TNF ordination analyses (Supplementary Fig. S3). When unrelated bacterial MAGs from other phyla were included, some phages were compositionally closer to non-host bacterial genomes than to their experimentally confirmed hosts. Thus, although compositional signals may remain informative in some contexts, TNF similarity alone is insufficient as a criterion for robust host assignment. The same concern applies to coverage-based binning, because lytic phages, induced prophages, and extracellular viral particles usually do not follow the abundance profiles of their bacterial hosts [19]. Because viral sequences were intentionally enriched for compositional similarity to the corresponding MAGs, this analysis should be interpreted as a stress test of MAG quality metrics under a challenging contamination scenario rather than as a model of random or typical viral contamination.

We then tested whether standard MAG quality metrics can detect the incorporation of non-host viral sequences. We selected 1000 high-quality non-Actinobacteriota MAGs from HRGM2 and added Actinobacteriophage-derived sequences with similar TNF to the MAGs [18] at total viral lengths of approximately 10, 50, 100, 150, or 200 kbp (Fig. 2a). The modified MAGs were re-evaluated using CheckM [11] and CheckM2 [13] (Fig. 2b–e). CheckM completeness estimates changed minimally even when approximately 200 kbp of viral sequence was added, whereas CheckM2 showed slightly larger but still limited bidirectional shifts (Fig. 2b and d). Contamination increased with the amount of viral sequence added, but most modified MAGs remained below the commonly used 5% threshold. In total, only five CheckM-evaluated and two CheckM2-evaluated MAGs exceeded this value across all simulations (Fig. 2c and e). To account for differences in MAG genome size, we additionally performed simulations in which viral sequences corresponding to approximately 1%, 5%, 10%, 15%, or 20% of the original MAG length were incorporated. These proportional simulations showed trends consistent with those based on fixed viral sequence lengths, with contamination estimates increasing with the proportion of incorporated viral sequences while remaining below 5% for the vast majority of MAGs even at the highest incorporation levels (Supplementary Fig. S4). Thus, substantial TNF-matched, non-host viral sequences can be incorporated into otherwise high-quality and unrelated MAGs while remaining largely invisible to conventional completeness and contamination estimates. However, this sampling scheme was intentionally designed to create a compositionally challenging contamination scenario and was not intended to approximate random viral incorporation into MAGs.

**Figure 2:**
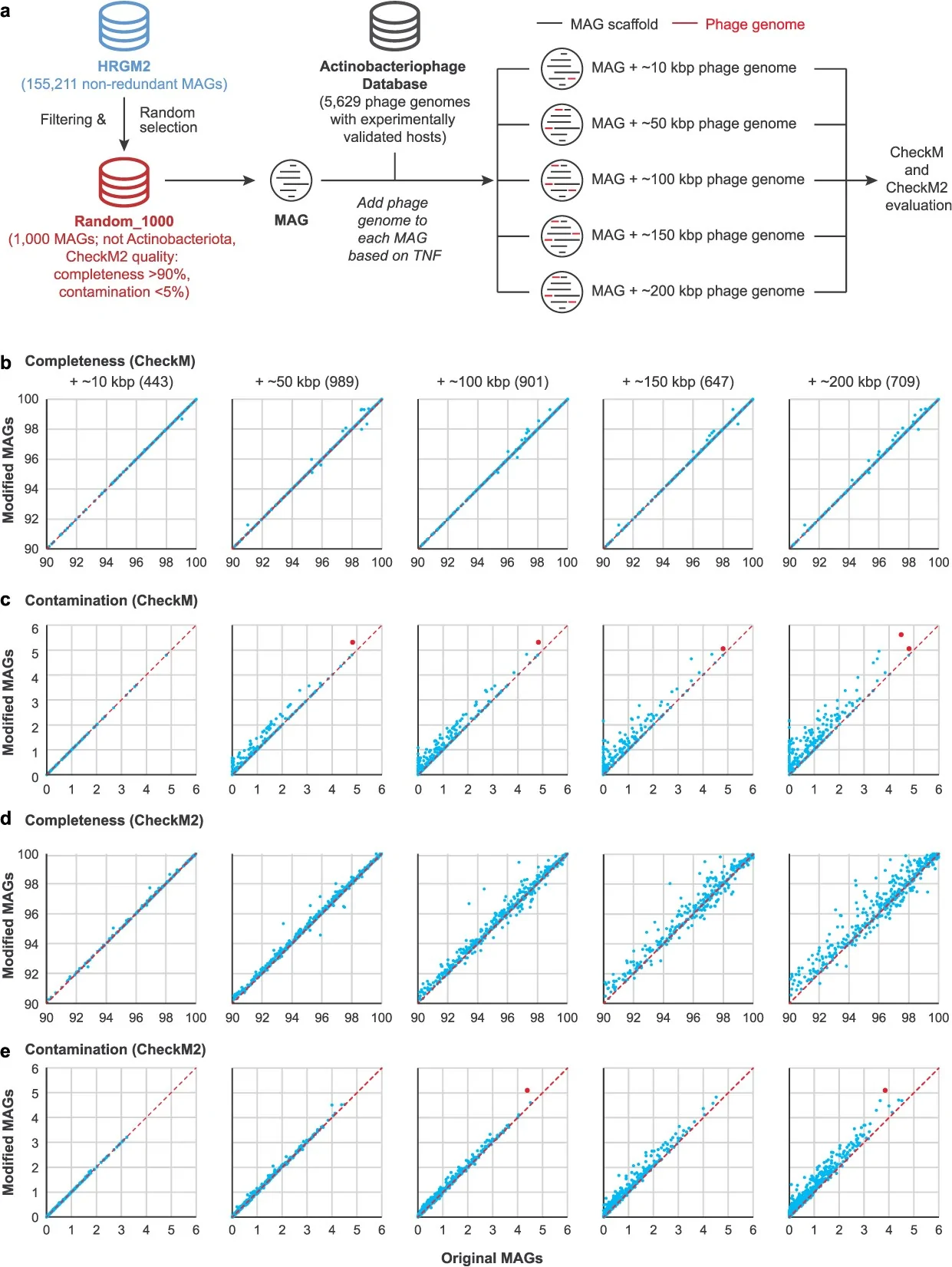
Incorporation of compositionally similar but non-host viral scaffolds into MAGs minimally affects completeness and contamination estimates. (a) Selection of high-quality bacterial MAGs and Actinobacteriophage genomes used for simulation. A total of 1000 high-quality bacterial MAGs (completeness ≥90% and contamination ≤5% by CheckM2), excluding Actinobacteriota, were randomly selected from the HRGM2 genome collection [9]. Actinobacteriophage genomes were obtained from the Actinobacteriophage Database [18] and used as viral sequence sources. For each MAG, viral genomic sequences with predefined total lengths (~10, 50, 100, 150, or 200 kbp) were incorporated based on TNF similarity. Some MAGs were excluded from downstream analyses because no suitable viral sequences with sufficiently similar TNF profiles could be identified (see Supplementary material). (b–e) Comparisons of completeness (b and d) and contamination (c and e) between original and modified MAGs using CheckM (b and c) and CheckM2 (d and e). In panels (b–e), each point represents one MAG, and the numbers of original–modified MAG pairs are indicated in brackets. The dashed red line indicates *y* = *x*. In panels (c and e), the five and two modified MAGs with contamination estimates exceeding 5% are highlighted with red circles.

These errors can propagate because public MAGs are increasingly used as reference resources for CRISPR spacer searches, prophage interpretation, viral homology searches, and machine-learning-based host prediction. A misbinned scaffold can therefore be reused repeatedly as supporting evidence across independent analyses. The problem also extends beyond phages, because plasmids, genomic islands, integrative elements, and other mobile genetic elements may violate the same binning assumptions, making scaffold identity more uncertain than the apparent quality of the host MAGs.

To assess CRISPR- and virus-related scaffolds in high-quality MAGs, we assessed the MAGs from the HRGM2 database [9]. Among 74 362 scaffolds containing Cas-associated CRISPR arrays, 6705 (9.0%) contained only 1–5 non-CRISPR genes, whereas 6037 (8.1%) and 61 620 (82.9%) contained 6–10 and ≥ 11 non-CRISPR genes, respectively (Supplementary Table 1). Among 188 323 virus-related scaffolds, 84 011 (44.6%) were predicted to be entirely viral. Among the remaining 104 312 provirus-containing scaffolds, 6542 (6.3%), 9762 (9.4%), and 88 008 (84.4%) contained 1–5, 6–10, and ≥11 non-viral genes, respectively (Supplementary Table 1).

Together, our analyses argue for a shift from MAG-level confidence to scaffold-aware validation in phage–host inference. We do not suggest that MAG-based predictions should be abandoned. Instead, host links inferred from MAG-derived CRISPR spacers, viral scaffolds, or viral–host sequence similarity should be treated as provisional unless supported by scaffold-level evidence. Practical validation should exclude CRISPR-only scaffolds lacking host genomic context, distinguish non-integrated viral scaffolds from candidate prophages, require virus–host junctions or sufficient host-flanking sequence for prophage-based assignment, and test whether the genes encoded by the non-CRISPR or non-viral regions are taxonomically consistent with the corresponding MAG, preferably at family-level resolution or finer. A stepwise workflow is provided in the Supplementary Information. We recognize that such taxonomy-based validation may not always be feasible when flanking genes encode hypothetical proteins or cannot be classified at sufficient resolution. When these criteria cannot be evaluated or are not met, MAG-based host assignments should be supported by independent evidence, such as multiple concordant spacer matches, long-read linkage, Hi-C contacts, co-occurrence profiles, or ideally experimental validation. As MAG repositories expand, scaffold-level validation will be essential for preventing binning errors from propagating into ecological and evolutionary interpretations of phage–host interactions.

## Supplementary Material

Supplementary_material_wrag276

## Data Availability

No original data were generated in this study. All datasets analyzed are publicly available, and source information is provided in the Supplementary material.
