# Supplementary material for: Scaffold-level validation is needed for phage–host inference from metagenome-assembled genomes
Source: ISME J. 2026 Nov 4;20(1):wrag276. doi: 10.1093/ismejo/wrag276 (PMC13768751; doi:10.1093/ismejo/wrag276)
Supplement: Supplementary file 1 [file supplementary_material_wrag276.zip › SI.pdf]

Supplementary Information

for

Scaffold-level validation is needed for phage–host inference from metagenome-assembled genomes

Ling Yuan (袁凌)[1], Karthik Anantharaman[2], Bin Ma (马斌)[3,4],

Antonio Pedro Camargo[5,*], LinXing Chen (陈林兴)[1,*]

[1] State Key Laboratory of Advanced Environmental Technology, School of Environment, University of Science and Technology of China, Hefei, China

[2] Department of Bacteriology, University of Wisconsin-Madison, Madison, WI, USA

[3] State Key Laboratory of Soil Pollution Control and Safety, Zhejiang University, Hangzhou, 310058, China

[4] Zhejiang Provincial Key Laboratory of Agricultural, Resources and Environment, College of Environmental and Resource Sciences, Zhejiang University, Hangzhou, 310058, China

[5] Department of Biochemistry, Institute of Chemistry, University of São Paulo, São Paulo, SP 05508-060, Brazil

[*]Corresponding author:

Antonio Pedro Camargo, antonio@iq.usp.br

LinXing Chen, linxingchen@ustc.edu.cn

Methods

TNF-based comparison of CRISPR repeat regions and the corresponding whole bacterial genomes.

To compare sequence composition between whole bacterial genomes and CRISPR repeat regions, we performed a TNF–based ordination analysis. We identified a total of 271 single-scaffold MAGs (Supplementary Table 2) from the HRGM2 datasets [1], and the CRISPR-Cas systems were predicted from them. Specifically, we first predicted the CRISPR repeat regions using PILER-CR version 1.06 with default parameters [2]. Then, the protein-coding genes within 2,000 bp upstream and downstream of the predicted repeat regions were searched for cas proteins using hmmsearch against the TIGRFAM HMM database with suggested threshold scores. If at least one Cas protein was detected within this initial 2,000 bp flanking region, the search window was extended to 10,000 bp upstream and downstream of the repeat region to identify additional Cas proteins associated with the same CRISPR-Cas locus. Repeat regions with at least one associated Cas protein were considered putative CRISPR-Cas systems. The Cas protein-encoding region was defined as the genomic span encompassing all identified Cas genes associated with a given repeat array, whereas the full CRISPR-Cas system region was defined as the continuous genomic span extending from the outermost boundary of the CRISPR repeat array to the outermost associated Cas gene. Thus, the 2-kb and 10-kb flanking windows were used only for Cas-gene identification and were not themselves used as the sequence regions for TNF calculation.

For each bacterial genome and its predicted CRISPR-Cas system, canonical tetranucleotide frequencies were calculated for four sequence types: (1) the whole genome, (2) the CRISPR repeat region predicted by PILER-CR, (3) the genomic span encompassing all associated Cas protein-encoding genes, and (4) the full CRISPR-Cas system region spanning both the repeat array and its associated Cas genes. All possible 4-mers composed of A, C, G, and T were considered, and each k-mer was collapsed with its reverse complement into a single canonical representation. Tetranucleotide counts were normalized by the total number of valid 4-mers in each sequence to obtain relative frequency profiles. No minimum length filtering was applied to CRISPR repeat array regions to retain short but compositionally informative sequences.

Pairwise compositional dissimilarities between sequences were calculated using Euclidean distance on the normalized TNF vectors. PCoA analysis was then performed on the resulting distance matrix using classical multidimensional scaling to project sequences into a low-dimensional compositional space. The proportion of variance explained by each axis was calculated from the eigenvalues of the centered distance matrix.

Although we focused on high-quality single-scaffold MAGs to minimize uncertainties associated with genome binning, future analyses using complete isolate genomes or long-read-resolved metagenome-assembled genomes will further evaluate the extent and generality of local genomic composition variation.

TNF-based comparison of experimentally validated actinobacteriophage–host genome pairs.

To evaluate whether viral genomes and their hosts exhibit systematic differences in TNF composition, we focused on actinobacteriophages that have experimentally validated host assignments. Host–phage relationships were obtained from the Actinobacteriophage Database [3], in which phage–host pairs are supported by isolation and infection experiments, thereby avoiding uncertainties associated with computational host prediction using bioinformatic tools.

We first identified the four host genera within the Actinobacteriophage Database (https://phagesdb.org/) that had the largest numbers of sequenced actinobacteriophage genomes. The corresponding host genomes were retrieved from NCBI when available. Among these four genera, only *Streptomyces* was represented by a sufficiently large number of publicly available host genomes with paired phage genomes to enable genome-level compositional analysis. Specifically, a total of 12 *Streptomyces* host genomes and 151 corresponding *Streptomyces*-infecting phage genomes were included (Supplementary Table 3).

We also searched NCBI using the keyword “E. coli phage” for isolated phage genomes of *E. coli*, and the corresponding host genomes were manually confirmed according to the NCBI descriptions of each phage genome. The host and isolated phage genomes were subsequently downloaded from NCBI when available, resulting in 7 *E. coli* and 87 phage genomes (Supplementary Table 4).

In addition, we added single-scaffold bacterial MAGs, the same ones we used in the CRISPR TNF-based analyses (Supplementary Table 2), to the *Streptomyces* and phage genome set, or the *E. coli* and phage genomes set, after removing the ones belonging to the same phylum.

For each host genome, scaffolds with a minimum length of 5 kbp were retained to minimize compositional noise associated with short sequences. Genome-level TNF profiles were then calculated by pooling canonical tetranucleotide counts from all retained scaffolds within each host genome, followed by normalization to relative frequencies. Thus, scaffolds were effectively weighted according to their sequence lengths rather than averaged

equally. For phages, TNF was calculated individually for each phage genome, with each genome treated as an individual analytical unit. TNF was computed as relative frequencies of canonical tetranucleotides, in which each 4-mer was merged with its reverse complement, and only unambiguous nucleotides (i.e., A, C, G, and T) were considered. Pairwise compositional distances between host and phage TNF profiles were calculated using Euclidean distance, and the resulting distance matrix was subjected to principal coordinates analysis (PCoA). Host and phage genomes were projected into a shared ordination space to visualize compositional relationships.

Evaluation of the incorporation of compositionally similar but non-host viral sequences into MAGs.

To assess the impact of co-binned and unrelated viral sequences on the completeness and contamination of MAGs, we performed two simulations and evaluations, including (1) co-binning random viral sequences into 1000 MAGs, and (2) co-binning random viral sequences into the E. coli K12 MG1655 genome.

We first simulated the co-binning of random viral sequences into multiple MAGs. Briefly, 1,000 bacterial MAGs, not from Actinobacteriota and with >90% completeness and <5% contamination (evaluated by checkM2), were randomly selected from the 155,211non-redundant genome collection of HRGM2 [1]. The information on the 1000 selected MAGs is available in Supplementary Table 5. The viral sequences were from the Actinobacteriophage Database (https://phagesdb.org/data/), which contains 5,629 genomes (downloaded on December 12, 2025) with experimentally validated hosts [3].

TNF of each Actinobacteriophage genome was calculated, and the average TNF of all scaffolds in each MAG was computed to represent the compositional profile of the corresponding MAG. Canonical TNF was used, in which each 4-mer was merged with its reverse complement, resulting in a 136-dimensional feature vector for each genome. For each MAG, the cosine similarity was calculated between its TNF profile and those of all Actinobacteriophage genomes. Based on these similarities, the top 100 most similar Actinobacteriophage genomes were selected as candidate viruses for that MAG. The Actinobacteriophage sequences were then stochastically introduced into each MAG by sampling from the candidate pool using a similarity-weighted scheme, in which the sampling probability of each Actinobacteriophage was proportional to a softmax transformation of its cosine similarity score. This approach preferentially selects viruses with TNF profiles more similar to the target MAG while retaining stochasticity in the selection process. For each MAG, one or more Actinobacteriophage genomes were added such that the total length of the introduced viral sequences fell within the defined range, i.e., 10 kbp, 50 kbp, 100 kbp, and 200 kbp, within a tolerance of ±10 kbp. Each viral Actinobacteriophage was added at most once to a given MAG but could be reused across different MAGs. This procedure generated a set of simulated MAGs with controlled levels of viral sequence incorporation while preserving compositional similarity between host and viral sequences.

To further account for differences in genome size among MAGs, we performed an additional simulation in which the amount of incorporated viral sequence was defined relative to the size of each MAG rather than as a fixed absolute length. The same 1,000 MAGs, Actinobacteriophage dataset, TNF-based candidate selection, and similarity-weighted sampling procedure described above were used. For each MAG, Actinobacteriophage genomes were added at four levels corresponding to approximately 1%, 5%, 10%, 15%, and 20% of the original MAG length. Because complete Actinobacteriophage genomes were incorporated without truncation, the exact target length could not always be achieved. We therefore allowed the total length of incorporated viral sequences to deviate by up to 10% from the target viral length (i.e., a relative tolerance of ±10% of the target). Multiple similarity-weighted combinations of candidate Actinobacteriophage genomes were sampled, and among combinations satisfying the specified length range, the combination with a total length closest to the target was retained. Each Actinobacteriophage genome was incorporated at most once into a given MAG but could be reused across different MAGs. MAGs for which no combination of candidate Actinobacteriophage genomes satisfied the specified length range were excluded from the corresponding comparison. This complementary simulation allowed us to evaluate the effects of viral sequence incorporation at comparable relative levels across MAGs of different genome sizes.

For all the original MAGs and genomes and the simulated genomes, we evaluated their quality using CheckM version 1.2.4 [4] with the “lineage_wf” mode and CheckM2 version 0.1.3 [5] with the “predict” mode, and the values of completeness and contamination were compared accordingly.

Quantification of genomic context on CRISPR-containing scaffolds in HRGM2

To evaluate the extent of scaffold-level genomic context supporting CRISPR-based phage–host assignments, we examined CRISPR-containing scaffolds in the HRGM2 genome collection [1]. Only scaffolds ⩾1 kbp were included. CRISPR repeat arrays and associated Cas genes were identified as described above in the section “**TNF-based comparison of CRISPR repeat regions and the corresponding whole bacterial genomes**.” Briefly, CRISPR-containing scaffolds were classified according to whether they contained repeat arrays without

associated Cas genes (repeat-only regions), repeat arrays associated with one or more Cas genes (repeat+Cas regions), or both types of regions on the same scaffold (Supplementary Table 1).

We first quantified the distribution of these scaffold types across HRGM2 MAGs and across all scaffolds ⩾1 kbp. Because CRISPR spacer-based phage–host inference requires evidence that a repeat array is associated with a CRISPR–Cas system, subsequent assessment of scaffold-level genomic context focused on scaffolds containing at least one repeat+Cas region. This included scaffolds containing only repeat+Cas regions as well as scaffolds containing both repeat-only and repeat+Cas regions.

To quantify genomic context independent of the CRISPR–Cas system itself, predicted CRISPR repeat regions were removed from these scaffolds and protein-coding genes were predicted from the remaining sequences using Prodigal version2.6.3 with the parameter "-m -p meta" [6]. Predicted Cas genes were then excluded, and all remaining protein-coding genes were considered non-CRISPR genes. For scaffolds containing multiple CRISPR regions, all identified repeat regions were removed before gene prediction, and genes from the resulting sequence fragments were subsequently reassigned to and summarized for their original scaffold. Scaffolds were grouped according to the number of remaining non-CRISPR genes (1–5, 6–10, or ⩾11).

Quantification of host genomic context on viral scaffolds in HRGM2 MAGs

To quantify the extent of host genomic context retained on virus-related scaffolds in existing MAGs, we analyzed high-quality bacterial MAGs (with completeness ⩾ 90% and contamination ⩽5% evaluated by CheckM) from the HRGM2 genome collection [1]. Viral sequences were identified following our previously described procedure [7]. In brief, all scaffolds with a minimum length of 10,000 bp were first analyzed using geNomad version 1.11.1 (end-to-end mode) [8], and the putative viral sequences were retained using conservative criteria (virus_score ⩾ 0.80, n_hallmarks ⩾ 1, mark_enrichment ⩾ 1.5, and fdr ⩽ 0.05). Only sequences passing these criteria were subsequently analyzed using VirSorter2 version 2.2.4 (--keep-original-seq --include-groups dsDNAphage,NCLDV,RNA,ssDNA,lavidaviridae) [9], and those with a VirSorter2 score ⩾ 0.5 were retained. The retained sequences were then processed using CheckV version 1.0.3 (end_to_end mode) to identify and remove host regions flanking predicted proviruses [10]. The resulting viral sequences were combined, and the extracted proviral sequences were additionally reanalyzed using CheckV to obtain quality information for the viral fragments. All resulting viral sequences were subsequently reanalyzed using VirSorter2 (--seqname-suffix-off --viral-gene-enrich-off --provirus-off --prep-for-dramv --include-groups dsDNAphage,NCLDV,RNA,ssDNA,lavidaviridae). Sequences were retained if they contained at least one predicted viral gene or, for sequences without predicted viral genes, if they contained no predicted host genes, had a VirSorter2 score ⩾ 0.95, or encoded more than two viral hallmark genes. Virus-related scaffolds were classified as either pure viral scaffolds, for which the entire scaffold was identified as viral, or provirus-containing scaffolds, for which a predicted proviral region occupied only part of the scaffold.

Pure viral scaffolds were considered to contain no non-viral genomic context. For each provirus-containing scaffold, the coordinates of all predicted proviral regions were determined based on the prediction results from CheckV [10]. When multiple proviral regions occurred on the same scaffold, overlapping or adjacent regions were merged before subsequent analysis. All sequences outside the resulting proviral regions were then extracted as non-viral regions. Protein-coding genes were predicted from these non-viral regions using Prodigal version2.6.3 with the parameter "-m -p meta" [6], and the total number of non-viral genes was determined for each scaffold.

Virus-related scaffolds were subsequently grouped according to the amount of non-viral genomic context retained on the same scaffold: 0 non-viral genes (pure viral scaffolds), 1–5 non-viral genes, 6–10 non-viral genes, or ⩾11 non-viral genes. The numbers and proportions of scaffolds in each category were calculated to quantify the extent to which viral scaffolds incorporated into high-quality MAGs retained scaffold-intrinsic genomic context that could be used to independently evaluate their association with the corresponding MAG.

Stepwise workflow for scaffold-level validation of MAG-based phage–host prediction

To facilitate practical implementation of scaffold-level validation, we propose a stepwise workflow for evaluating whether scaffolds containing CRISPR-related or viral regions retain sufficient host genomic context and whether that context is taxonomically consistent with the corresponding MAG. This workflow is intended as an additional validation step after MAG reconstruction and phage–host inference, rather than as a replacement for existing genome-binning or taxonomic-classification procedures.

First, CRISPR-related or viral regions on each scaffold should be identified and their genomic coordinates recorded. These regions should then be excluded from the subsequent host-context analysis so that taxonomic inference is based only on the remaining putative non-CRISPR or non-viral sequences. Protein-coding genes can be predicted from the non-CRISPR or non-viral portions using a standard gene-calling tool such as Prodigal.

Genes overlapping the excluded regions should be omitted from downstream taxonomic evaluation. Scaffolds with very little residual sequence or too few predicted protein-coding genes, for example, <5 genes, should be considered to have insufficient host genomic context.

Second, the predicted proteins from the non-CRISPR or non-viral sequences should be compared against a comprehensive taxonomically annotated reference database. For example, proteins predicted from GTDB genomes can be used to construct an MMseqs2 reference database, and significant matches can be mapped back to the taxonomy of their source genomes. Appropriate thresholds for sequence identity, alignment coverage, and statistical significance should be selected according to the intended taxonomic resolution. Taxonomic inference should rely on multiple informative non-CRISPR or non-viral genes rather than on a single protein match. When different genes support different taxonomic levels, assignment should be restricted to the lowest rank consistently supported by the available evidence.

Third, the taxonomic signal derived from the non-CRISPR or non-viral portion of each scaffold should be compared with the taxonomy of the corresponding MAG, which can be independently assigned using GTDB-Tk or an equivalent genome taxonomy framework based on the same reference release. A scaffold can be considered taxonomically concordant when multiple informative non-CRISPR or non-viral genes support a lineage consistent with the corresponding MAG, preferably at the family level or finer. A scaffold can be considered taxonomically discordant when multiple informative genes consistently support a lineage different from that assigned to the MAG. Scaffolds for which informative genes yield conflicting assignments should be treated as ambiguous, whereas scaffolds with too few informative non-CRISPR or non-viral genes or insufficiently resolved taxonomic annotations should be treated as having insufficient host context.

Taxonomic concordance provides scaffold-level support for a MAG-based host assignment, thus suggesting proof of a phage–host relationship. Conversely, taxonomic discordance indicates that the scaffold assignment and associated host inference should be re-evaluated. Importantly, a lack of taxonomic resolution should not be interpreted as evidence of misbinning. Scaffolds with ambiguous or insufficient host context should instead be treated as unresolved, and the corresponding MAG-based host assignments should remain provisional unless supported by independent evidence, such as multiple concordant CRISPR spacer matches, phage-host junctions, long-read linkage, Hi-C contacts, co-occurrence profiles, or experimental validation.

Supplementary Note: Broader implications of scaffold-level uncertainty in MAG-based phage–host inference

Our analyses highlight a distinction that is often blurred in genome-resolved metagenomics analyses: MAG-level quality and scaffold-level correctness are related but not equivalent. Completeness and contamination estimates are valuable for assessing whether a bacterial or archaeal genome bin (or MAG) has been substantially recovered, but they primarily rely on conserved cellular marker genes. They therefore provide limited information about whether individual scaffolds encoding CRISPR arrays, viral regions, plasmid sequences, genomic islands, or other mobile genetic elements are correctly assigned to the organism represented by the MAG [11]. This limitation becomes consequential when MAGs are used not only as reconstructed genomes, but also as reference frameworks for inferring virus–host links.

CRISPR-associated scaffolds represent one major source of uncertainty. CRISPR repeat–spacer arrays can vary extensively among closely related strains because new spacers are acquired in response to recent exposure to viruses and plasmids. This biological variability can fragment CRISPR loci in short-read assemblies, producing scaffolds that contain only repeat–spacer arrays or incomplete CRISPR–Cas regions. In addition, CRISPR–Cas systems can behave as accessory elements and undergo horizontal transfer [12–14]. As a result, the local sequence composition of CRISPR-associated regions may deviate from that of the broader host genome. These features explain why CRISPR repeat–spacer arrays can occupy TNF space distinct from their host genomes (Figs. S1 and S2). If such a scaffold is incorrectly binned into a MAG, any spacer match derived from it may falsely link a virus to the taxon represented by that MAG. Therefore, CRISPR-based host prediction should distinguish isolated CRISPR-array scaffolds from scaffolds that contain cas genes and sufficient host genomic context.

Viral scaffolds present a related but distinct problem. Phage genomes do not necessarily share the tetranucleotide composition of their experimentally confirmed hosts. Their genome composition reflects viral evolutionary history, replication strategy, gene content, genome architecture, and host adaptation, but these factors do not guarantee host-like composition. In our analyses (Fig. S3), some phages were closer in TNF space to unrelated bacterial genomes than to their known hosts. This does not mean that compositional information is useless, but it shows that compositional similarity should not be treated as sufficient evidence for host linkage. Coverage-based information can be misleading as well. During lytic infection, intracellular viral replication and extracellular virion accumulation may produce viral coverage far above that of the host chromosome. Conversely, prophages in low-copy or partially induced states may display discordant coverage patterns across samples. These dynamics reduce the reliability of co-abundance as an independent signal for host assignment. In

summary, due to these natural features of phages, the two principal dimensions used in conventional binning, i.e., sequence composition and sequencing coverage, may both be systematically violated for viral sequences.

The simulation results explain why such errors can persist in apparently high-quality MAGs (Fig. 2). When TNF-matched, non-host viral sequences were added to high-quality MAGs, CheckM and CheckM2 generally retained high-quality classifications, and contamination estimates usually remained below the 5% threshold. This outcome is expected because most viral sequences do not introduce redundant conserved cellular marker genes, which are the primary basis for marker-gene-based contamination estimates. In this sense, the issue is not simply that MAG quality metrics are imperfect, but that they are poorly aligned with the specific problem of viral scaffold misassignment. A MAG can therefore be high quality for the purpose of representing a cellular genome while still being unreliable as a source of host evidence for a particular viral or CRISPR-containing scaffold.

These findings have practical consequences for phage–host prediction workflows, and the broader concern extends beyond phage–host prediction. Plasmids, genomic islands, integrative conjugative elements, transposon-rich regions, and other mobile genetic elements are also prone to scaffold-level misbinning in short-read metagenomic assemblies. As public genome catalogs continue to grow, they are increasingly reused as reference datasets for ecological and evolutionary inference. Hidden scaffold-level errors can therefore propagate from genome repositories into downstream analyses, including horizontal gene transfer inference, auxiliary metabolic gene interpretation, mobile element ecology, and machine-learning-based prediction frameworks. Scaffold-aware validation should therefore be considered a necessary complement to existing MAG quality standards, especially in studies focused on viruses and other mobile genetic elements.

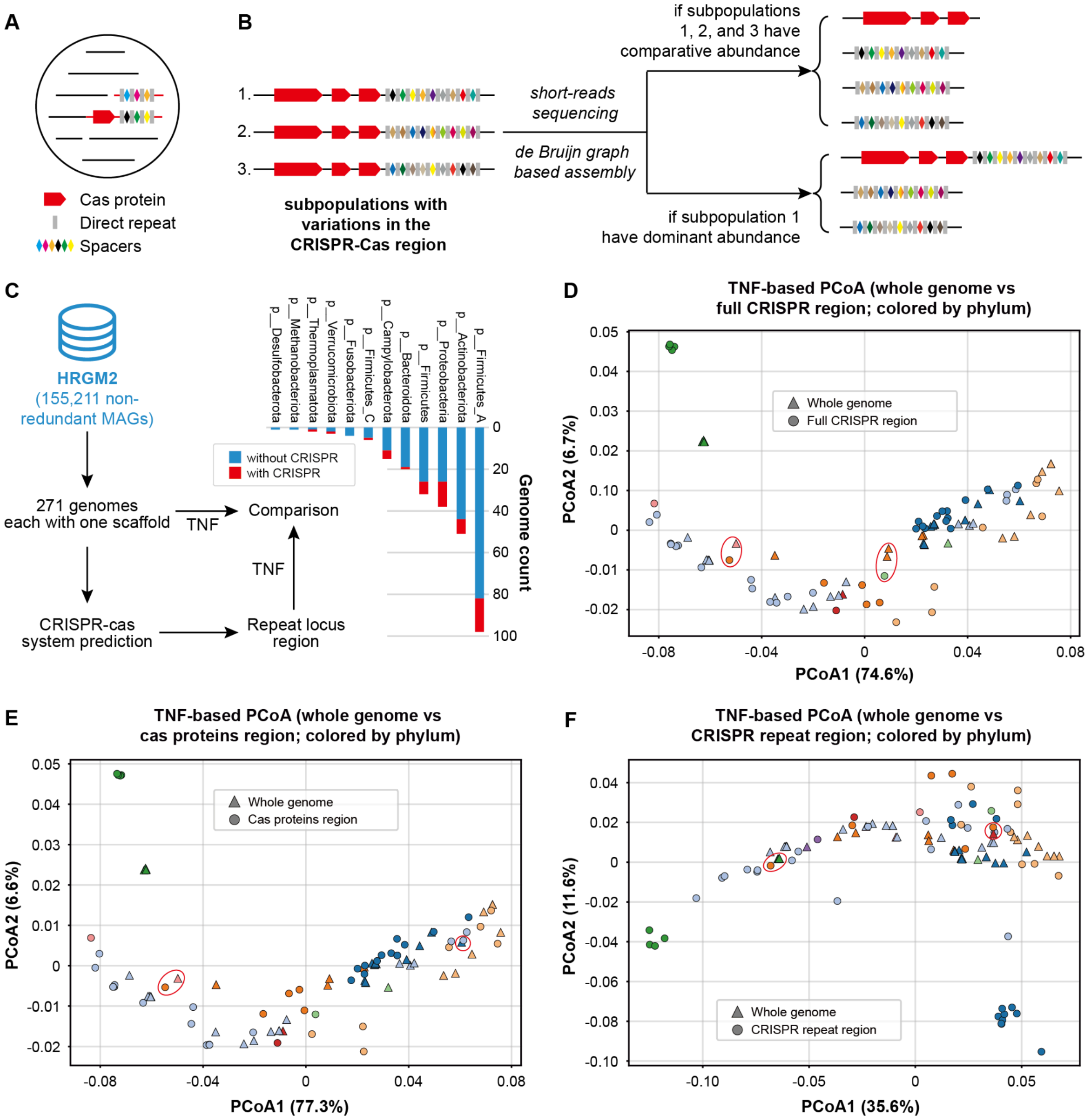

Fig. S1 | CRISPR–Cas–encoding scaffolds in metagenomic analyses. (A) Schematic illustration of the two principal modes by which CRISPR–Cas–encoding scaffolds are incorporated into metagenome-assembled genomes (MAGs). (B) Representative scenarios illustrating how genomic fragments are generated during metagenomic assembly of short paired-end reads using de Bruijn graph–based assemblers. (C) Identification and characterization of CRISPR–Cas systems within 271 single-scaffold MAGs from the HRGM2 dataset (Supplementary Table 2). TNF–based PCoA (based on a cosine distance matrix) comparing sequences of the whole genomes against (D) the full CRISPR-Cas system regions, (E) the Cas protein-coding regions, and (F) the CRISPR repeat regions. Red circles indicate sequences of whole genomes and CRISPR-related regions from different phyla, which could be binned together if they had comparable sequencing depth and were presented in the same community. Note that close proximity in TNF space does not necessarily indicate close taxonomic relatedness, as genomes from distantly related taxa can exhibit similar oligonucleotide composition.

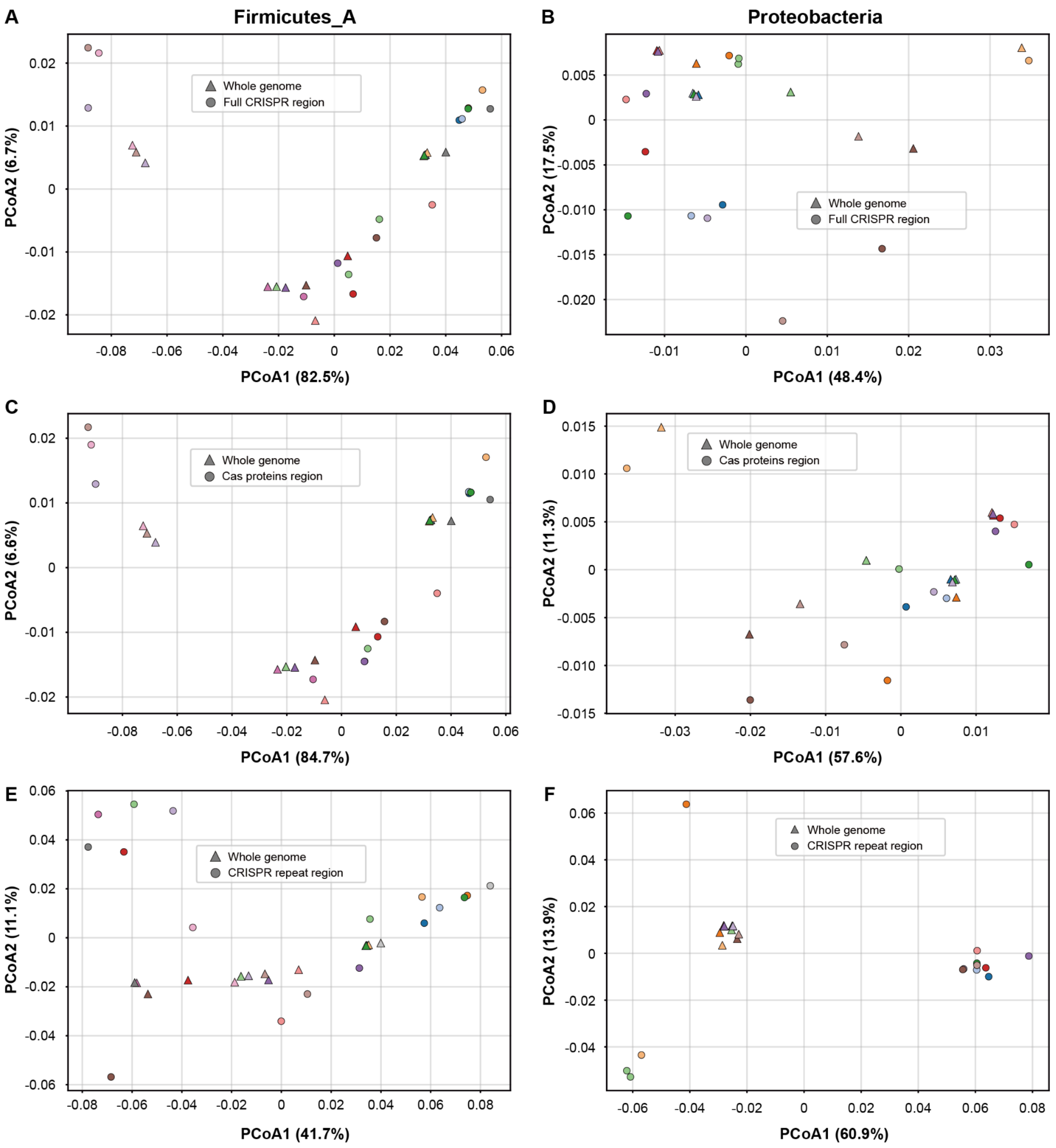

Fig. S2 | TNF–based PCoA comparison of CRISPR-related sequences against the whole genomes at the genome level. Comparison of the whole genomes against (A) and (B) the full CRISPR-Cas system regions, (C) and (D) the Cas protein regions, (E) and (F) the CRISPR repeat regions, for those from the phylum of (A) (C) (E) Firmicutes_A and (B) (D) (F) Pseudomonadota.

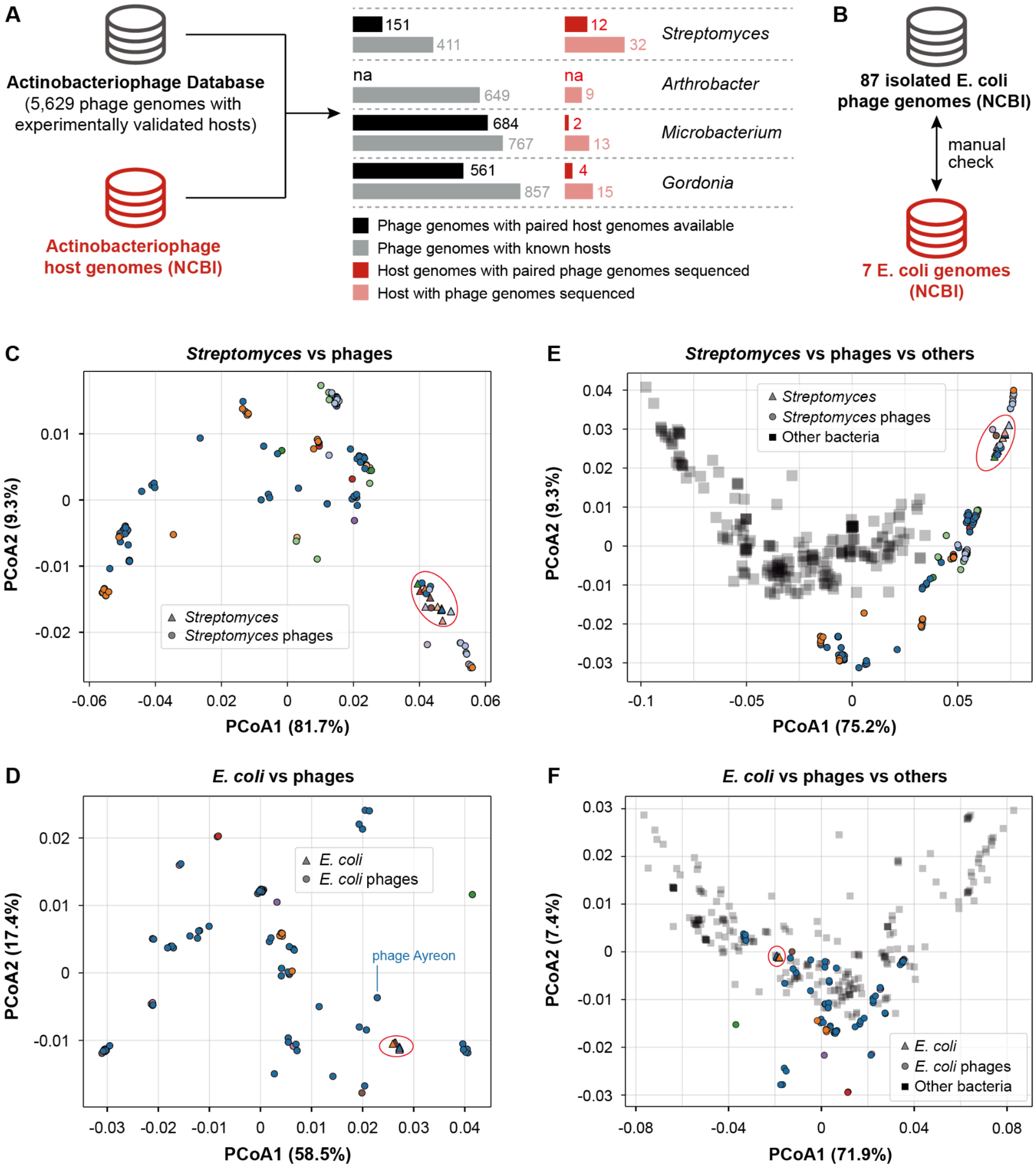

Fig. S3 | Phage genomes exhibit TNF compositions distinct from their bacterial hosts. (A) Overview of experimentally validated actinobacteriophage–host associations curated from the Actinobacteriophage Database [3]. The information on host genera and sequenced infecting phage genomes is included in Supplementary Table 3. (B) Manual retrieval of genomes for *E.coli* and their isolated phages from NCBI (Supplementary Table 4). Principal coordinates analysis (PCoA) of genome-level TNF profiles for host genomes (scaffolds ⩾ 5 kbp) and their experimentally validated infecting phage genomes of (C) *Streptomyces* spp. and (D) *E. coli*. TNF-based PCoA analysis of (E) *Streptomyces* and their phage genomes with 220 non-Actinobacteriota single-scaffold bacterial MAGs (Supplementary Table 2), and (F) *E. coli* and their phage genomes along with 233 non-Pseudomonadota single-scaffold bacterial MAGs (Supplementary Table 2). In (C)-(F), host genomes were indicated by triangles, phage genomes by circles, and other bacterial MAGs by squares. Host–phage pairs are indicated using identical colors. The host genomes are highlighted by the red circles.

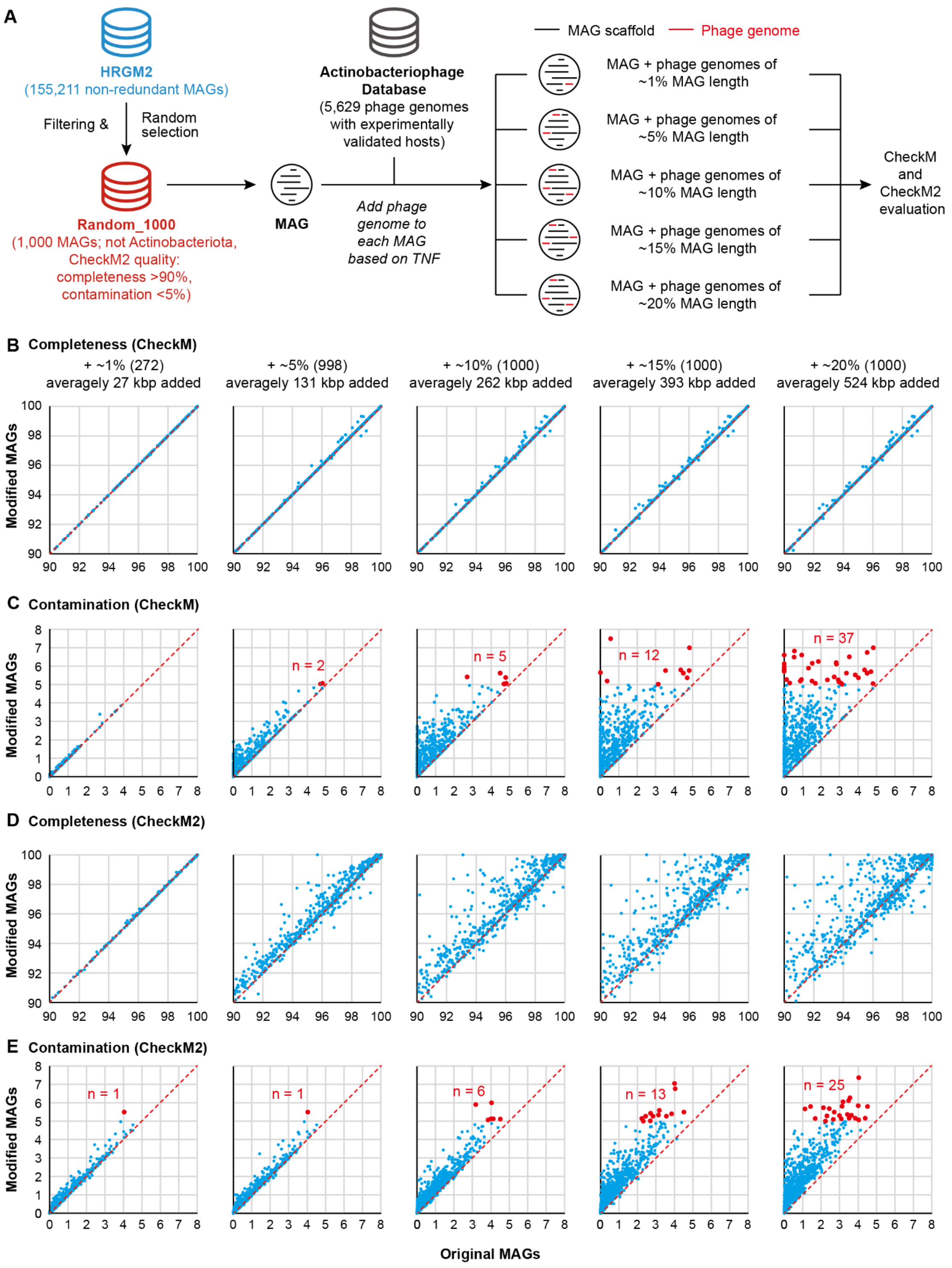

Supplementary Fig. S4 | Incorporation of compositionally similar but non-host viral scaffolds at different proportions of MAG length. (A) Selection of high-quality bacterial MAGs and Actinobacteriophage genomes used for simulation. A total of 1,000 high-quality bacterial MAGs (completeness ⩾ 90% and contamination ⩽ 5% by CheckM2), excluding Actinobacteriota, were randomly selected from the HRGM2 genome collection [1]. Actinobacteriophage genomes were obtained from the Actinobacteriophage Database [3] and used as viral sequence sources. For each MAG, viral genomic sequences corresponding to approximately 1%, 5%, 10%, 15%, or 20% of the original MAG length were incorporated based on TNF similarity. Some MAGs were excluded from individual simulations because no suitable combination of viral sequences satisfying the specified target range could be identified (see Supplementary Information). (B)–(E) Comparisons of completeness (B and D) and contamination (C and E) between original and modified MAGs using CheckM (B and C) and CheckM2 (D and E). In panels (B)–(E), each point represents one MAG, and the numbers of original–modified MAG pairs and the average lengths of incorporated viral sequences are indicated above each plot. The dashed red line indicates $y = x$. In panels (C) and (E), modified MAGs with contamination estimates exceeding 5% are highlighted with red circles, and the numbers are shown.

References

1. Ma J et al. A human gut metagenome-assembled genome catalogue spanning 41 countries supports genome-scale metabolic models. *Nat Microbiol* 2026;**11**:317–334.
2. Edgar RC. PILER-CR: fast and accurate identification of CRISPR repeats. *BMC Bioinformatics* 2007;**8**:18.
3. Russell DA, Hatfull GF. PhagesDB: the actinobacteriophage database. *Bioinformatics* 2017;**33**:784–786.
4. Parks DH et al. CheckM: assessing the quality of microbial genomes recovered from isolates, single cells, and metagenomes. *Genome Res* 2015;**25**:1043–1055.
5. Chklovski A et al. CheckM2: a rapid, scalable and accurate tool for assessing microbial genome quality using machine learning. *Nat Methods* 2023;**20**:1203–1212.
6. Hyatt D et al. Prodigal: prokaryotic gene recognition and translation initiation site identification. *BMC Bioinformatics* 2010;**11**:119.
7. Chen L et al. Animal-associated jumbo phages as widespread and active modulators of gut microbiome ecology and metabolism. *Sci Adv* 2026;**12**:eaeb6265.
8. Camargo AP et al. Identification of mobile genetic elements with geNomad. *Nat Biotechnol* 2023. https://doi.org/10.1038/s41587-023-01953-y
9. Guo J et al. VirSorter2: a multi-classifier, expert-guided approach to detect diverse DNA and RNA viruses. *Microbiome* 2021;**9**:37.
10. Nayfach S et al. CheckV assesses the quality and completeness of metagenome-assembled viral genomes. *Nat Biotechnol* 2020. https://doi.org/10.1038/s41587-020-00774-7
11. Chen L-X et al. Accurate and complete genomes from metagenomes. *Genome Res* 2020;**30**:315–333.
12. Pinilla-Redondo R et al. CRISPR-Cas systems are widespread accessory elements across bacterial and archaeal plasmids. *Nucleic Acids Res* 2022;**50**:4315–4328.
13. Makarova KS et al. Evolutionary classification of CRISPR–Cas systems: a burst of class 2 and derived variants. *Nat Rev Microbiol* 2019;**18**:67–83.
14. Makarova KS et al. An updated evolutionary classification of CRISPR-Cas systems including rare variants. *Nat Microbiol* 2025;**10**:3346–3361.
